# Topology-driven discovery of transmembrane protein *S*-palmitoylation

**DOI:** 10.1016/j.jbc.2025.108259

**Published:** 2025-02-03

**Authors:** Michael T. Forrester, Jacob R. Egol, Sinan Ozbay, Farrah D. Waddell, Rohit Singh, Purushothama Rao Tata

**Affiliations:** 1 Division of Pulmonary, Allergy and Critical Care Medicine, Duke University School of Medicine, Durham, North Carolina, USA; 2 Department of Cell Biology, Duke University School of Medicine, Durham, North Carolina, USA; 3 Duke Regeneration Center, Duke University School of Medicine, Durham, North Carolina, USA

**Keywords:** gradient boosting, machine learning, *S*-acylation, *S*-palmitoylation, transmembrane protein

## Abstract

Protein *S*-palmitoylation is a reversible lipophilic posttranslational modification regulating diverse signaling pathways. Within transmembrane proteins (TMPs), *S*-palmitoylation is implicated in conditions from inflammatory disorders to respiratory viral infections. Many small-scale experiments have observed *S*-palmitoylation at juxtamembrane Cys residues. However, most large-scale *S*-palmitoyl discovery efforts rely on trypsin-based proteomics within which hydrophobic juxtamembrane regions are likely underrepresented. Machine learning—by virtue of its freedom from experimental constraints—is particularly well suited to address this discovery gap surrounding TMP *S*-palmitoylation. Utilizing a UniProt-derived feature set, a gradient-boosted machine learning tool (TopoPalmTree) was constructed and applied to a holdout dataset of viral *S*-palmitoylated proteins. Upon application to the mouse TMP proteome, 1591 putative *S*-palmitoyl sites (*i.e.* not listed in SwissPalm or UniProt) were identified. Two lung-expressed *S*-palmitoyl candidates (synaptobrevin Vamp5 and water channel Aquaporin-5) were experimentally assessed, as were three Type I transmembrane proteins (Cadm4, Chodl, and Havcr2). Finally, TopoPalmTree was used for the rational design of an *S*-palmitoyl site on KDEL-Receptor 2. This readily interpretable model aligns the innumerable small-scale experiments observing juxtamembrane *S*-palmitoylation into a proteomic tool for TMP *S*-palmitoyl discovery and design, thus facilitating future investigations of this important modification.

Protein *S*-palmitoylation is a reversible posttranslational modification that allows for hydrophobic “tuning” of specific protein regions. For membrane-associated proteins such as the Ras family of GTPases, *S*-palmitoylation facilitates proper membrane localization and signaling (1). Within transmembrane proteins (TMPs), *S*-palmitoylation is believed to modulate protein conformation relative to the lipid bilayer (2), ultimately contributing to diverse biological outputs from extracellular signal transduction to endocytic sorting. In the case of pathogenic coronaviruses, *S*-palmitoylation of the viral Spike protein (a TMP) is essential for proper envelope geometry and viral replication (3, 4).

Gradient-boosted trees (GBTs) are a type of supervised iterative machine learning that employs gradient descent to improve the performance of successive trees (5). These flexible algorithms can handle different types of data, be tuned to minimize overfitting, and maintain a structure amenable to model interpretation. Within molecular biology, gradient-boosted machines have been employed for predictive models of *O*-phosphorylation (6, 7), protein-small molecule (8), and -DNA (9) interactions. Gradient boosted trees, in particular, frequently outperform other methods such as Naïve Bayes, support vector machines, and logistic regression (10) as manifested by the widespread success of GBTs in machine learning competitions.

Despite the biological relevance of TMP *S*-palmitoylation, our knowledge about molecular determinants of *S*-palmitoylation has primarily relied on small-scale studies focused on individual proteins. Early experiments suggested a narrow range of distance from the lipid bilayer in dictating *S*-palmitoylation of an individual Cys residue on ER protein p63, whereas adjacent amino acids were less critical (11). A globally applicable motif for TMP *S*-palmitoylation has not been found (12) and typical molecular cues for *S*-palmitoylation, such as *N*-myristoylation or C-terminal isoprenylation, do not appear operative on TMPs. Despite the enigmatic nature of TMP *S*-palmitoylation, this modification has been shown to dictate the localization of TMPs to specific membrane microdomains (13, 14), receptor internalization from the plasma membrane (15, 16), proper tilting of transmembrane domains relative to the lipid bilayer (17, 18) and assembly into organized protein complexes (2, 12).

Gradient-boosted trees are known to excel with complex mixtures of categorical and continuous features, yet GBTs remain to be utilized for *S*-palmitoyl site discovery (19). Further, most established *S*-palmitoyl inference tools primarily rely on sequence information (20, 21, 22) rather than the topological features supported by small-scale experiments. Motivated by statistical (23) and experimental (24, 25) investigations suggesting unique physiochemical determinants for TMP *S*-palmitoylation, we have sidestepped the traditional one-size-fits-all model and constructed a TMP-specific algorithm for *S*-palmitoyl discovery.

## Results

### Juxtamembrane regions are underrepresented in large-scale *S*-palmitoyl data

The prevailing strategy for large-scale *S*-palmitoyl discovery is some form of *S*-acyl derivation followed by bottom-up proteomics. Examples include “click-able” lipid probes amenable to cycloaddition-based capture (26, 27, 28, 29, 30, 31) and hydroxylamine-based *S*-acyl cleavage followed by either thiol biotinylation (32, 33, 34) or direct capture onto a solid-phase matrix (35, 36, 37). Shared between these approaches is the use of proteolysis (*i.e.*, trypsinization) and liquid chromatography-mass spectrometry (LC-MS) to identify putative *S*-acylated sites. Trypsin is an efficacious and highly specific protease, yet it provides poor recovery for certain regions of mammalian proteomes (38, 39) leading to the notion of proteomic “tunnel vision” (40). For example, transmembrane proteins (TMPs) exhibit regions of elevated hydrophobicity that are underrepresented in bottom-up proteomics due to their relative resistance to trypsinization and poor aqueous solubility (41, 42). However, 20 to 30 percent of mammalian open reading frames encode TMPs, many of which are signaling receptors and clinically relevant pharmacological targets. Given the propensity of Cys residues to undergo *S*-palmitoylation in these hydrophobic juxtamembrane regions, we hypothesized that *S*-palmitoyl datasets derived primarily from bottom-up proteomics might encompass only a small fraction of *bona fide S*-palmitoylated sites.

**Figure 1:**
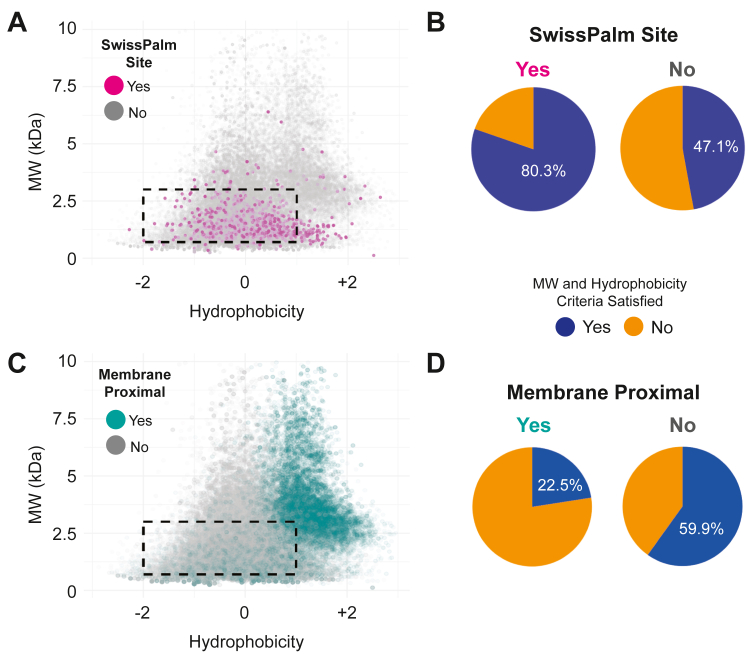
**Experimental “detectability” of TMP *S*-palmitoyl sites.** The murine transmembrane proteome was subjected to *in silico* trypsinization, followed by labeling of Cys-containing peptides based on whether they are (*A* and *B*) reported as *S*-palmitoylated in SwissPalm (*magenta*) or (*C* and *D*) within 20 amino acids of a transmembrane domain and thus proximal to the lipid bilayer (*teal*). This concept of “membrane proximity” is frequently observed in small scale studies of *S*-palmitoylation and thus underpins the hypothesis that juxtamembrane locations are *S*-palmitoyl “hot spots”. The dashed rectangle represents a general detectible region for bottom-up proteomics of 700 to 3000 Da and mean Kyte-Doolittle hydrophobicity of −2 to +1. The pie charts indicate the fraction of Cys-containing peptides that fall within the detectable range based on whether the Cys sites are (*B*) reported in SwissPalm or (*D*) juxtamembrane in location.

To evaluate this question, the mouse transmembrane proteome was subjected to *in silico* trypsinization followed by biophysical peptide analysis. Of 63,380 Cys-containing peptides, 4357 match to reported *S*-acylated sites in SwissPalm with 955 of these sites being located on a TMP. The physical properties of all Cys-containing peptides were plotted concerning molecular weight and mean hydrophobicity (based on Kyte-Doolittle values). As shown in Figure 1, *A* and *B*, the majority (80.3%) of TMP *S*-palmitoyl sites in SwissPalm fall into the ideal range of detectability defined as MW 700 to 3000 Da and mean hydrophobicity of −2 to +1. However, categorizing the same 63,380 Cys residues by membrane proximity—defined as being within 20 amino acids of a transmembrane domain—reveals that only 22.5% of membrane-proximal Cys-containing peptides fall within this ideal range (Fig. 1, *C* and *D*). These findings suggest that membrane-proximal *S*-palmitoyl “hot spots” may be underrepresented in high throughput experiments, the vast majority of which employ trypsin-based bottom-up proteomics. While experimentally tailored proteolysis can improve TMP recall in bottom-up proteomics, *in silico* approaches can completely circumvent these peptide-level biophysical constraints.

### Establishment of a high-fidelity *S*-palmitoyl TMP training dataset

**Figure 2:**
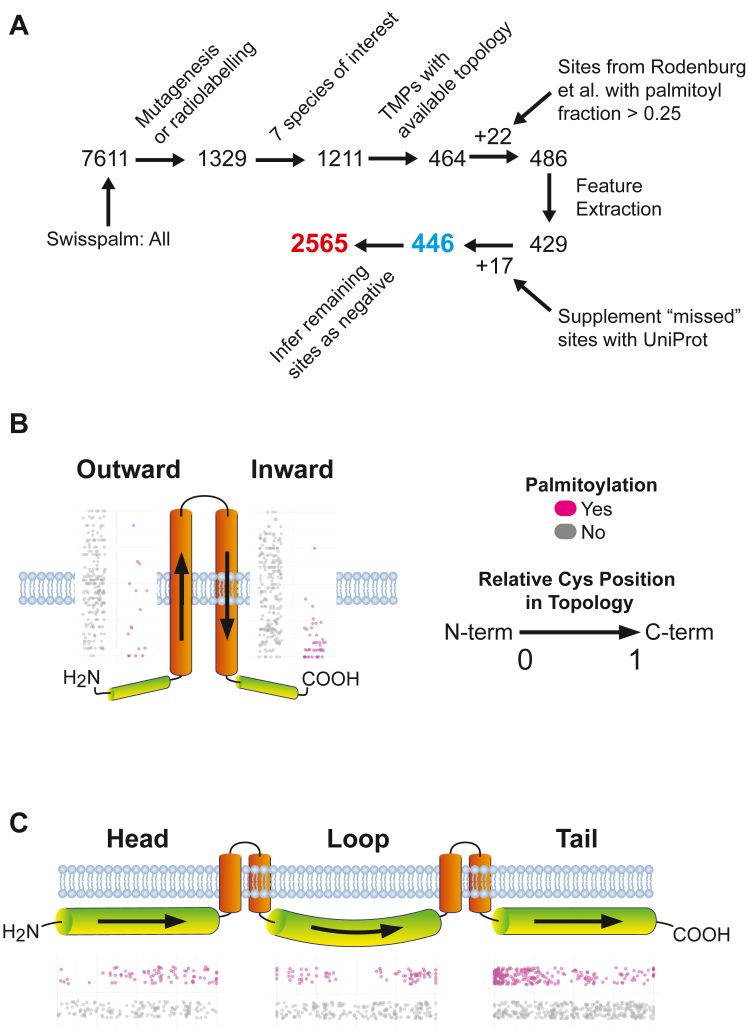
**Establishing the training dataset and exploration of S-palmitoyl site locations.***A*, schematic of training data isolation and pre-processing. Data were filtered to include sites confirmed by experiments utilizing site-directed mutagenesis or palmitate radiolabeling. The seven included species were *H. sapiens* (58.15%), *M. musculus* (23.65%), *R. norvegicus* (11.62%), *A thaliana* (2.59%), *S. cerevisiae* (1.93%), mutant proteins from Rodenburg *et al.* (0.73%) and *B. taurus* (0.60%). A total of 429 sites were suitable for feature extraction, with an additional 17 positive sites supplemented onto those proteins from UniProt-derived data (*i.e.*, S-palmitoyl sites not present in SwissPalm). Within proteins containing these 446 positive sites, the remaining 2565 Cys residues were inferred as negative class. Locations of S-palmitoyl sites within (*B*) transmembrane and (*C*) cytoplasmic regions. Shown alongside each region is a jitterplot of relative Cys location in Topology where 0 and 1 represent the N- and C-terminal ends of each region. Outward and inward transmembrane domains are oriented with their C-terminal sides in the extracellular and intracellular regions, respectively.

To isolate a training dataset with a reliable and relatively complete representation of TMP *S*-palmitoyl sites, large-scale proteomics data was set aside in favor of data primarily obtained by two rigorous methods: site-directed mutagenesis and ^3^H-palmitate radiolabeling. Filtering SwissPalm for these methodological criteria resulted in a preliminary training dataset of 464 *S*-palmitoyl sites including 68 (15%) and 287 (62%) sites confirmed by some variety of click-based lipid labeling or hydroxylamine-based acyl exchange, respectively. Additional data (4.9% of the positive class) was obtained from Rodenberg *et al.* (24), with every Cys site at least 25% *S*-palmitoylated when expressed in mammalian cells and quantitated using native LC-MS considered “positive”. These include 17 sites from claudin-3, 4 sites from bacteriorhodopsin, and one site from pH-gated potassium channel KcsA. After filtering and collecting available topological data from UniProt, 446 sites of *S*-palmitoylation were obtained with a class imbalance of 6.75:1 in favor of non-*S*-palmitoyl sites (Fig. 2*A*). The dataset was composed of multi- and single-pass TMPs (Fig. S1*A*) with a broad distribution of *S*-palmitoyl sites across cytoplasmic and transmembrane regions (Fig. S1*B*).

### Exploratory data analysis implicates the cytoplasmic-membrane interface in *S*-palmitoylation

Many experiments have confirmed TMP *S*-palmitoylation at juxtamembrane regions (2, 11, 12, 24, 43, 44). To gauge the validity of the training dataset and interrogate the relevance of this proposed feature, the training dataset was analyzed in terms of distance from the cytoplasmic-transmembrane interface. Measuring from the topological end, inward-oriented transmembrane domains showed median distances of 1 (mean 2.2) *versus* 10 (mean 9.6) amino acids for *S*-palmitoyl *versus* non-*S*-palmitoyl sites, respectively (Fig. 2*B*). For outward domains, median distances were four (mean 5.8) *versus* 10 (mean 10.1) amino acids from the topological start. Consistent with experimental observations, cytoplasmic *S*-palmitoyl sites exhibited general proximity to the lipid bilayer with median distances of 8.5 and 12 amino acids for cytoplasmic heads and tails, respectively (Fig. 2*C*). In contrast, their non-*S*-palmitoyl Cys counterparts had a median membrane distance of 151 and 116 amino acids for cytoplasmic heads and tails, respectively. These measurements align with the many small-scale observations of *S*-palmitoylation clustering near the transmembrane-cytoplasmic interface prompting us to incorporate such measurements as features in TopoPalmTree. Distance from the N- and C-termini may also be a determinant (both relative and absolute) (19). We hypothesized that these parameters would be critical elements for the model.

Local physiochemical parameters are also important to the feature engineering process. In the case of S-palmitoylation, no universal “motif” has been captured through large-scale analyses. However, adjacent basic residues are consistently noted near S-palmitoyl sites suggesting that regional trends in polarity, charge state and hydrophobicity may play roles. Capturing such information is generally performed by establishing a “window” of amino acids flanking each Cys residue. Given the remarkable proximity of S-palmitoyl sites as noted earlier (25% percentile of cytoplasmic S-palmitoyl sites only three amino acids from the transmembrane domain), we sought to capture local information without introducing noise. Windows used for S-palmitoyl inference range from 5 to 16 amino acids (19) and we, therefore, settled on the low-end allowing information to be obtained from 10 total adjacent amino acids (5 on each side).

### *S*-palmitoylation is associated with regional trends in hydrophobicity

Given their juxtamembrane nature, one would expect *S*-palmitoyl hot spots to be in areas with changing hydrophobicity as the polypeptide spans from cytoplasmic to transmembrane regions and *vice versa*. To capture this biophysical feature, mean hydrophobicity was calculated for each Cys using a window sequence of 5 amino acids in N- and C-terminal directions constituting absolute (sum) and gradient (difference) in values. A positive gradient represents increasing hydrophobicity towards the C-terminus and *vice versa*. Within transmembrane domains, total hydrophobicity was higher and lower for *S*-palmitoyl sites on inward- and outward-oriented regions, respectively (Fig. S2*A*). Hydrophobicity gradients were more pronounced for *S*-palmitoyl compared to non-*S*-palmitoyl sites, consistent with *S*-palmitoylation clustering at the cytoplasmic-membrane interface where hydrophobicity gradients would be expected to undergo large value swings (Fig. S2*B*). Within cytoplasmic regions, *S*-palmitoyl sites exhibited higher total hydrophobicity (Fig. S2*C*) along with higher gradients (Fig. S2*D*).

### Cysteine cooperativity and minor feature assignments

Sites of *S*-palmitoylation are often clustered with multiple adjacent Cys residues with evidence of cooperativity (24, 45) or acting as a barrier to de-palmitoylation (46). To build this phenomenon into our feature set, a window Cys scoring scheme was developed that provides points for adjacent Cys residues within the window sequence (Fig. S3*A*). Within the training dataset, *S*-palmitoyl sites showed a higher mean window Cys score (Fig. S3*B*) suggesting that *S*-palmitoyl cooperativity occurs in TMPs and allowing the model to exploit this biological feature.

During exploratory data analysis, a relative paucity of asparagine residues was noted in the windows of *S*-palmitoyl sites (Fig. S3, *C* and *D*). Interestingly, a similar trend was not observed for glutamine despite these amino acids having structurally similar side chains. Aside from relative asparagine depletion, polybasic residues were readily observed within the windows of cytoplasmic *S*-palmitoyl sites (Fig. S4). These are known to promote protein-membrane association (47) and *S*-palmitoylation (48) at least in part through decreasing the Cys thiol pKa (49, 50). Other window-based features incorporated into TopoPalmTree include polarity (51), net charge, aliphatic index, and transmembrane tendency (52).

### Training the gradient-boosted tree and validation with a viral *S*-palmitoyl dataset

**Figure 3:**
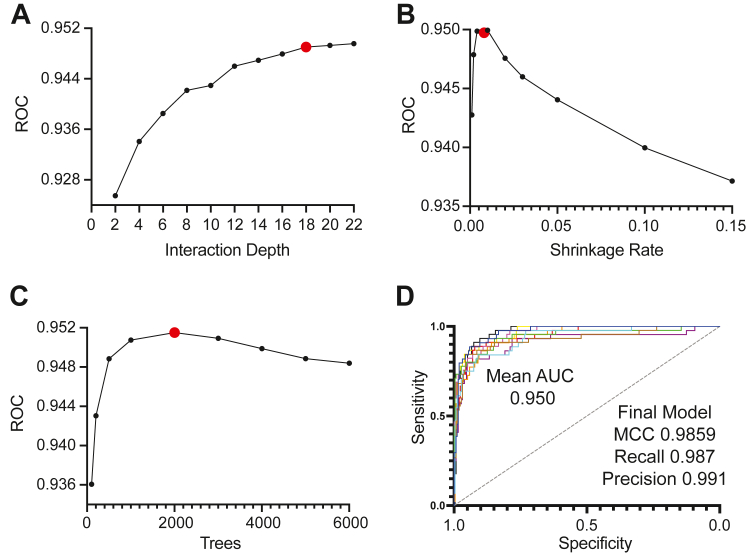
**Training and hyperparameter tuning of TopoPalmTree.** Shown on each y-axis are mean AUC values from the receiver operator curve *versus* (*A*) interaction depth, (*B*) number of trees, and (*C*) shrinkage rate. Chosen hyperparameters are highlighted in *red*. *D*, performance on 10-fold cross validation with chosen hyperparameters of interaction depth = 18, ntree = 2000, shrinkage = 0.08, and m.minobsinode = 5. Statistics relevant to the final model fitting are shown in (*D*).

The gradient-boosted tree (GBT) is a powerful machine learning technique with desirable properties such as model interpretability, flexibility with respect to types of tasks, automatic feature importance, and robust methods to prevent overfitting. Upon creation of 28 distinct features within the training data, TopoPalmTree was trained *via* the gbm package in R. Hyperparameter tuning was performed through grid search to optimize the interaction depth, shrinkage rate, and number of trees (Fig. 3, *A*–*C*). Model performance was relatively robust to hyperparameter choice, exhibiting high AUC on 10-fold cross-validation and performance metrics on final model fitting (Fig. 3*D*). Feature importance plotting (Fig. S5) confirmed the importance of Cysteine location, Cys score (*i.e.* cooperativity), hydrophobicity, and polarity for the model.

Many viral TMPs rely on *S*-palmitoylation during the viral infection cycle (4, 53). Given their reliance on host *S*-palmitoyl machinery (54) yet complete lack of sequence homology, viral *S*-palmitoyl TMPs represent an attractive holdout dataset to rigorously assess TopoPalmTree performance. Starting with SwissPalm and experimental criteria of point-mutation or ^3^H-palmitate radiolabeling utilization, 27 viral *S*-palmitoyl TMPs were identified. Another four and five were identified in UniProt and the primary literature (4), respectively. Of these 36 proteins, 30 had topological annotations available through UniProt. No more than three orthologues were allowed for each type of protein. Following feature extraction, this dataset provided 82 *S*-palmitoyl and 641 non-S-palmitoyl sites, respectively. Compared to the training dataset, the class imbalance of the viral holdout was slightly higher (8.81 *versus* 6.75).

**Figure 4:**
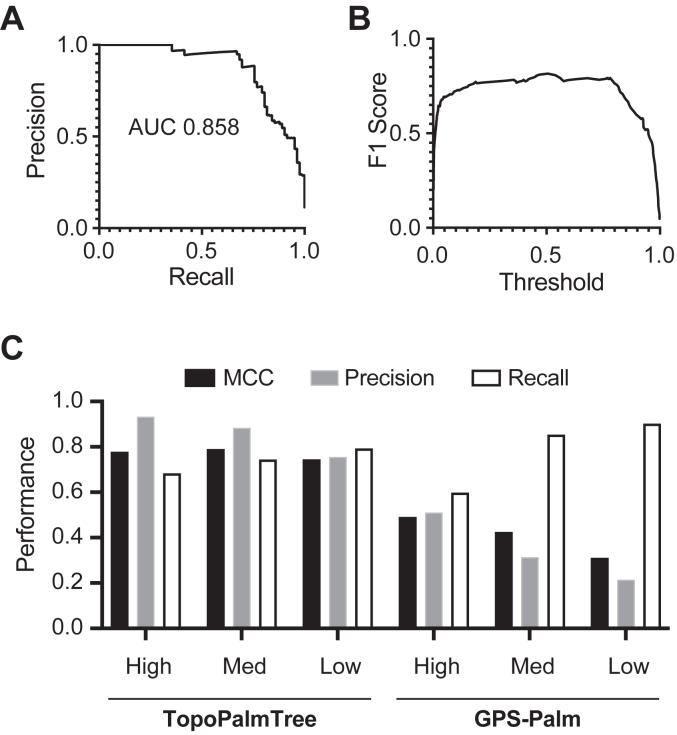
**TopoPalmTree validation with a holdout dataset and benchmarking to GPS-Palm.** A dataset of 30 viral *S*-palmitoylated proteins (containing 82 *S*-palmitoyl sites) was employed for holdout given complete lack of sequence similarity to training data. Performance shown as (*A*) precision-recall curve and (*B*) F1 score *versus* threshold. *C*, performance of TopoPalmTree at three different thresholds (Low: 0.25, Med: 0.50, High: 0.75) compared to GPS-Palm and the three available thresholds of Low, Med, High.

To avoid the beneficial effects of negative assignments on metrics such as accuracy, the AUC from precision *versus* recall was plotted over a continuous threshold range (Fig. 4*A*). The high AUC suggests that either precision or recall can be optimized with relatively little loss in the other metric’s performance. To better understand model performance across all threshold values, the harmonic mean of precision and recall (F1 score) was plotted against the threshold (Fig. 4*B*). This plot showed a relatively consistent F1 score of 0.8 suggesting stable performance across the range of thresholds. Utilizing the viral TMP dataset, TopoPalmTree’s performance was compared to GPS-Palm, a recently developed neural network model for *S*-palmitoyl prediction (20) showing even stronger performance than the widely utilized clustering-based tool CSS-Palm (55, 56). Comparing across three threshold values, TopoPalmTree exhibited a higher and more consistent Matthews Correlation Coefficient (MCC) than GPS-Palm (Fig. 4*C*). Improving recall *via* threshold lowering showed that TopoPalmTree’s precision remained relatively preserved, whereas GPS-Palm showed a marked deterioration in precision and MCC. The complete results of TopoPalmTree on the viral dataset are listed in Table S1.

### Application of TopoPalmTree to the murine transmembrane proteome

**Figure 5:**
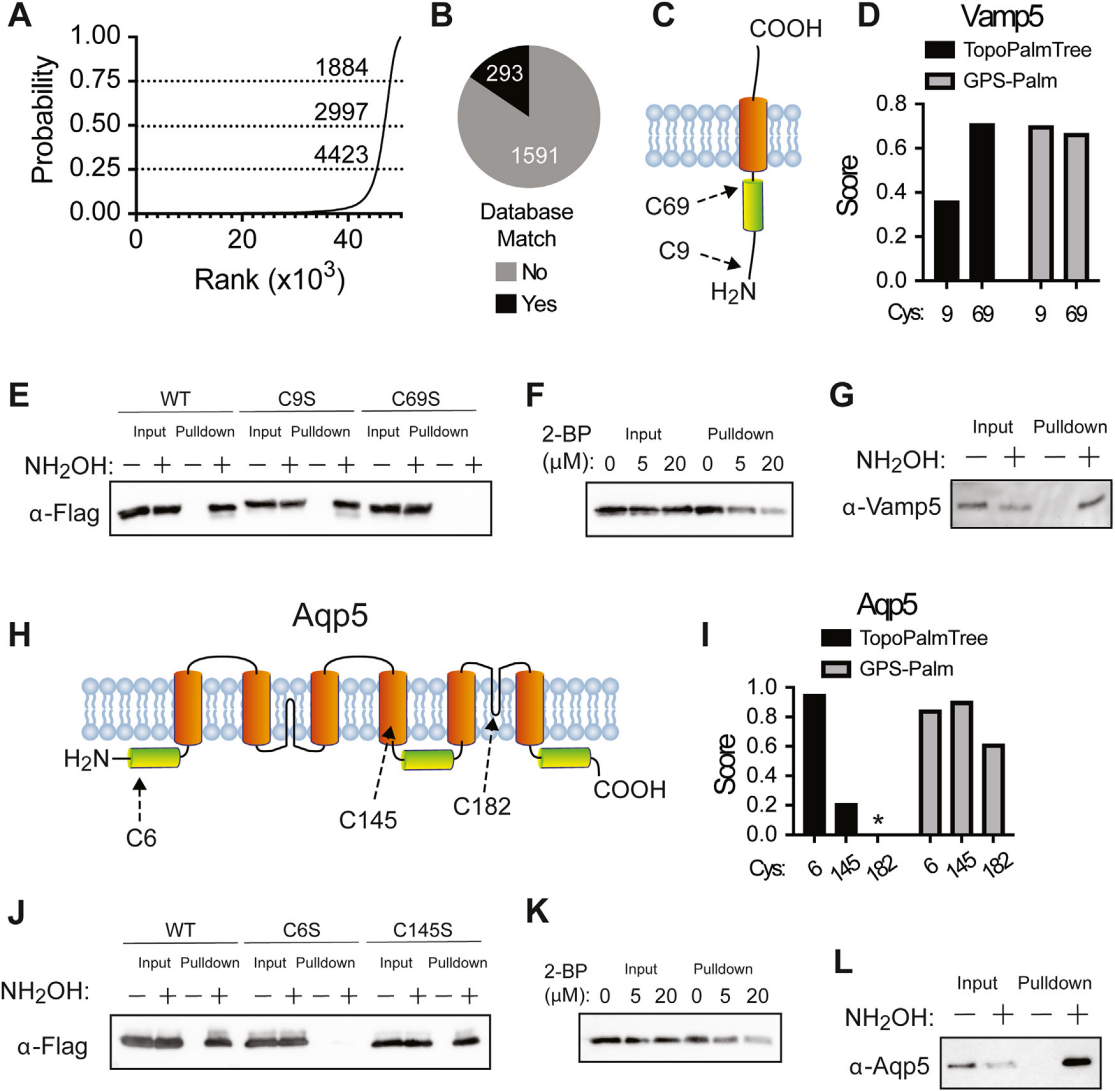
**Application of TopoPalmTree for *S*-palmitoyl site discovery.***A*, rank order plot of TopoPalmTree score for each Cys of the murine TMP proteome (49,828 total). Thresholds (dashed lines) are shown at 0.75 (high), 0.50 (medium), and 0.25 (low) with an associated number of inferred *S*-palmitoyl sites at each threshold value. *B*, pie chart of database site matches (UniProt and SwissPalm combined) from the high cutoff compared to the number of inferred sites that have not been reported in either database. *C*, schematic of Vamp5 topology. *D*, Vamp5 probability scores from TopoPalmTree *versus* GPS-Palm. *E*, Acyl-RAC of Vamp5-flag Cys mutants in HEK293 cells (n = 4). *F*, Acyl-RAC of Vamp5-flag from HEK293 cells co-treated with vehicle (DMSO) or 2-BP for 18 h (n = 3). *G*, Acyl-RAC of endogenous Vamp5 in murine lung (n = 3). *H*, schematic of Aqp5 topology. Shown are transmembrane domains (*orange*) and two intramembrane regions (*black*) that do not traverse the entire lipid bilayer. *I*, Aqp5 probability scores from TopoPalmTree *versus* GPS-Palm. Given the rare nature of intramembrane regions not represented in the training data, TopoPalmTree does not provide a probability score for Cys^182^ (asterisk). *J*, Acyl-RAC of Aqp5-flag Cys mutants in HEK293 cells (n = 4). *K*, Acyl-RAC of Aqp5-flag from HEK293 cells co-treated with DMSO or 2-BP for 18 h (n = 3). *L*, Acyl-RAC of endogenous Aqp5 in murine lung (n = 3).

After filtering the TMP proteome for suitable topological data, 5009 murine TMPs containing 49,828 Cys residues were amenable to inference by TopoPalmTree (Table S2). As shown in Figure 5*A*, the output from TopoPalmTree revealed 1884 Cys residues (3.8%) deemed high likelihood (probability > 0.75), whereas 45,405 Cys sites (91.1%) have probabilities below 0.25. These findings suggest that TopoPalmTree is discerning toward the positive (*S*-palmitoyl) class and aligns with intuitive expectations of *S*-palmitoylation being restricted to a minority of Cys residues. Of the 1884 putative *S*-palmitoyl Cys sites, 293 are reported (between UniProt and SwissPalm) leaving 1591 sites as novel putative sites of *S*-palmitoylation identified by TopoPalmTree (Fig. 5*B*).

### Experimental confirmation and discovery of *S*-palmitoyl sites in mouse lung

To examine the utility of TopoPalmTree to guide the discovery of *S*-palmitoylation, two candidate TMPs (Vamp5 and Aquaporin-5) were cloned and subjected to experimental analysis for *S*-acylation. As shown in Figure 5*C*, Vamp5 has two Cys residues: Cys9 located near the N-terminus and juxtamembrane Cys69 located 4 amino acids from the transmembrane domain. As shown in Figure 5*D*, TopoPalmTree showed a higher score for Cys69 compared to Cys9 (0.71 *versus* 0.36), while GPS-Palm exhibited a slightly higher score for Cys9 than Cys69 (0.70 *versus* 0.67). Within TopoPalmTree’s rank output, Cys69 and Cys 9 ranked 2045 and 3681 (out of 49,828), respectively. When subjected to experimental analysis for *S*-acylation by Acyl-RAC (36), Vamp5-transfected HEK293 cells exhibited hydroxylamine-dependent pulldown that was completely lost upon mutation of Cys69 to Serine (Fig. 5*E*). Pulldown was dose-dependently inhibited by 2-bromopalmitate (Fig. 5*F*) and observed endogenously in the murine lung (Fig. 5*G*). Notably, Cys69 is flanked by Arg residues and is essentially “lost” as a dipeptide when subjected to trypsinization. This likely explains why Vamp5 Cys69 has never been reported as a site of *S*-palmitoylation, yet it was readily identified by TopoPalmTree. Collectively these findings establish Cys69 as the site of *S*-palmitoylation on Vamp5 and demonstrate the utility of TopoPalmTree in identifying *S*-palmitoylation sites, particularly those that may be missed by bottom-up proteomics.

**Figure 6:**
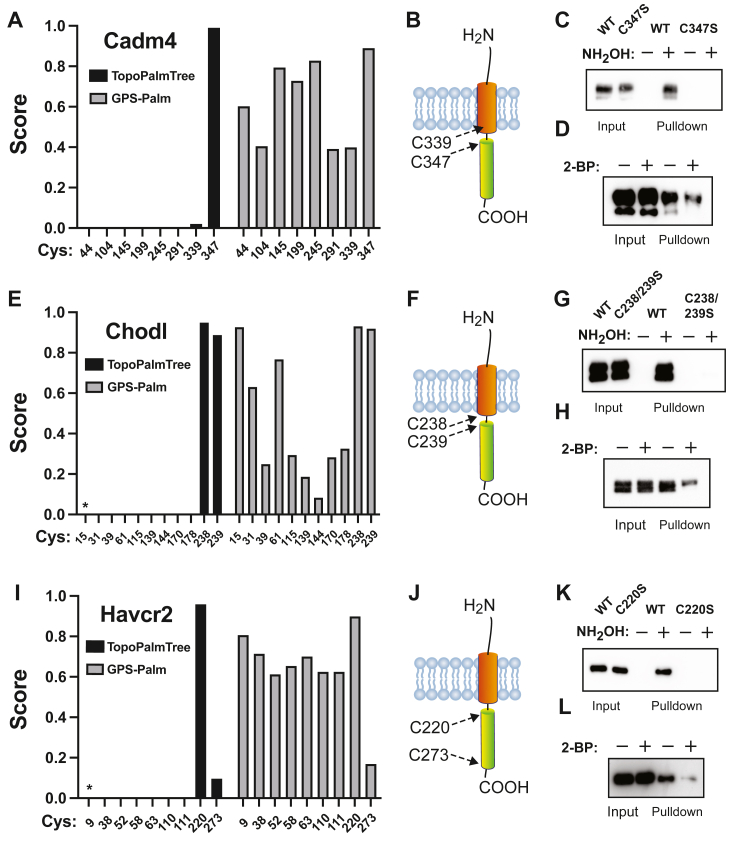
**Assessment of mouse Type I TMPs by Acyl-RAC.***A*, score outputs from TopoPalmTree compared to GPS-Palm for cell adhesion molecule 4 (Cadm4). *B*, schematic of Cadm4 with relative locations of Cys339 (within a transmembrane domain) and Cys347. *C*, mammalian expression plasmids containing Cadm4-flag WT *versus* C347S were transfected into HEK293 cells and subjected to Acyl-RAC in the presence or absence of hydroxylamine (n = 4). *D*, acyl-RAC of Cadm4-flag expressing HEK293 cells pre-treated with either vehicle (DMSO) or 20 μM 2-BP (n = 3). *E*, score outputs for chondrolectin (Chodl). *F*, schematic of Chodl highlighting Cys238 and Cys239 located at the interface of transmembrane and cytoplasmic domains. *G*, Chodl-flag WT *versus* C238/239S were expressed in HEK293 cells and subjected to Acyl-RAC (n = 3). *H*, Acyl-RAC of Chodl-flag WT expressing HEK293 cells pre-treated with either vehicle or 20 μM 2-BP (n = 3). *I*, score outputs for hepatitis A virus cellular receptor two (Havcr2). *J*, schematic of Havcr2 showing juxtamembrane Cys220 and C-terminal adjacent Cys273. *K*, Acyl-RAC of Havcr2-flag WT *versus* C220S expressing HEK293 cells (n = 3). *L*, Acyl-RAC of Havcr2-expressing HEK293 cells pre-treated with either vehicle or 40 μM 2-BP (n = 3). Asterisk (∗) indicates a Cys residue on a signal peptide, for which TopoPalmTree cannot provide an inference.

Another candidate *S*-palmitoyl TMP is Aquaporin-5 (Aqp5), a multi-pass TMP enriched in alveolar type 1 cells and is responsible for water transport across the alveolar membrane (57, 58). Aquaporin 5 has three Cys residues: cytoplasmic Cys6 located seven amino acids from the lipid bilayer, Cys145 located within a transmembrane region, and Cys182 located in a so-called “intramembrane” region buried inside the lipid bilayer yet not spanning the membrane (Fig. 5*H*). These intramembrane domains are present within only 1.1% of the murine proteome. Given their paucity and lack of representation in the training dataset, intramembrane Cys residues (*e.g.* Aqp5 Cys182) were omitted from inference by TopoPalmTree. As shown in Figure 6*I*, TopoPalmTree strongly favored Cys6 over Cys145 (score 0.95 *versus* 0.21). By rank order analysis, TopoPalmTree ranked Cys6 at 647 compared to Cys145 at 4775 (out of 49,828 total). In contrast, GPS-Palm suggested a slight preference for Cys145 (0.90) followed by Cys6 (0.84) and Cys182 (0.61). Of note, Aqp5 Cys6 was detected in a proteomic study of bovine lens (59) but has not been subjected to prediction methods or confirmatory studies such as site-directed mutagenesis. As shown in Figure 6*J*, HEK293 cells transfected with Aqp5 showed hydroxylamine-dependent pulldown by the Acyl-RAC assay with loss of signal upon mutation of Cys6 to Ser. Mutation of Cys145 to Ser had no effect, again demonstrating the superior performance of TopoPalmTree in identifying the correct site of *S*-palmitoylation. Pulldown of Aqp5 was dose-dependently inhibited by 2-bromopalmitate (Fig. 6*K*) and observed endogenously in murine lung (Fig. 5*L*). Similar to Vamp5, Aqp5 serves as another proof-of-concept that TopoPalmTree can recognize sites of TMP *S*-palmitoylation and facilitate targeted hypothesis testing.

### Experimental assessment of type I transmembrane proteins

Type I TMPs are abundant (>1400 in human proteome) and involved in a wide range of homeostatic and stress-responsive signaling functions. To further assess TopoPalmTree, three candidate Type I TMPs with at least one Cys site scoring > 0.75 were selected for experimental analysis. These include cell adhesion molecule 4 (Cadm4), chondrolectin (Chodl), and hepatitis A virus cellular receptor homolog 2 (Havcr2). Within Cadm4, the only high-scoring site is Cys347 located two amino acids in the cytoplasmic domain (Fig. 6, *A* and *B*). In contrast, a nearby potential site (Cys399) located seven amino acids into the transmembrane region was scored low by TopoPalmTree. Upon expression of Cadm4-flag into HEK293 cells, pulldown by Acyl-RAC was dependent on the presence of Cys347 (Fig. 6*C*) and antagonized by 2-BP (Fig. 6*D*). In the course of these experiments, Cadm4 Cys347 was independently discovered as an *S*-palmitoyl site regulating Cadm4 plasma membrane localization and nervous system myelination (60). Of note, the tryptic peptide containing Cys347 is 48 amino acids in length and includes the entire hydrophobic transmembrane region. Such a peptide is poorly suited for trypsin-based LC-MS thus going “undiscovered” when trypsin is used for protein digestion.

Chondrolectin is a 30 kD TMP involved in neurite growth and is dysregulated in spinal muscular atrophy (61, 62). As shown in Figure 6*E*, TopoPalmTree provided high scores for adjacent residues Cys238 and 239 located at the first positions of the cytoplasmic domain (Fig. 6*F*). Similar to Cadm4, *in silico* trypsinization reveals Cys239/239 resides on a 65 amino acid polypeptide containing the entire transmembrane region. Not surprisingly, these residues have not been observed in large-scale S-palmitoyl studies and are not known to undergo S-palmitoylation. Subjecting Chodl-flag to expression in HEK293 cells and Acyl-RAC revealed loss of pulldown with mutation of Cys238/239 to Ser (Fig. 6*G*) and partial blockade by treatment with 2-BP (Fig. 6*H*).

Hepatitis A virus cellular receptor homolog 2 (Havcr2) is a Type I TMP expressed in the adaptive immune system and plays broad roles in T-cell inhibition (63). This protein was selected for testing due to the presence of two cytoplasmic Cys residues worthy of experimental assessment: high-scoring Cys220 located just five amino acids into the cytoplasmic domain and low-scoring Cys273 located only eight amino acids from the C-terminus (Fig. 6*I* and *J*). Experimental assessment of Havcr2-flag by Acyl-RAC in HEK293 cells again showed loss of pulldown with mutation of Cys220 to Ser (Fig. 6*K*) and diminished pulldown in the presence of 2-BP (Fig. 6*L*). In the course of these experiments, human Havcr2 (TIM-3) was also reported to undergo S-palmitoylation on Cys296 adjacent to the C-terminus (64). Mouse and human Havcr2 are only 64% identical with the human orthologue and have several critical differences that may be relevant to S-palmitoylation. Human Havcr2 lacks Cys220 (His residue in this position) and contains an 11 amino acid C-terminal extension (including Cys296) not present in murine Havcr2. The low-scoring Cys273, however, is conserved between human and mouse Havcr2 with both our results and those of Zhang *et al.* (64) arguing that this site is not a target of S-palmitoylation. It is worth speculating that these differences in *S*-palmitoylation sites between human and mouse Havcr2 may underpin biological differences in adaptive immunity between these mammals.

It is also worth noting that the highest-scoring sites from GPS-Palm corresponded to the experimentally confirmed sites, thus showing a fair degree of model agreement for these three TMPs (Fig. 6, *A*, *E* and *I*) within the context of evaluating only the highest-scoring site. However, GPS-Palm trended towards less discriminatory scoring between sites, particularly for Havcr2 where 9 of the 10 Cys sites scored > 0.5. Collectively, these observations establish the utility of TopoPalmTree for discovering sites of *S*-palmitoylation, particularly those that are poorly suited for traditional bottom-up proteomics.

### Rational design of an *S*-palmitoyl site on KdelR2

**Figure 7:**
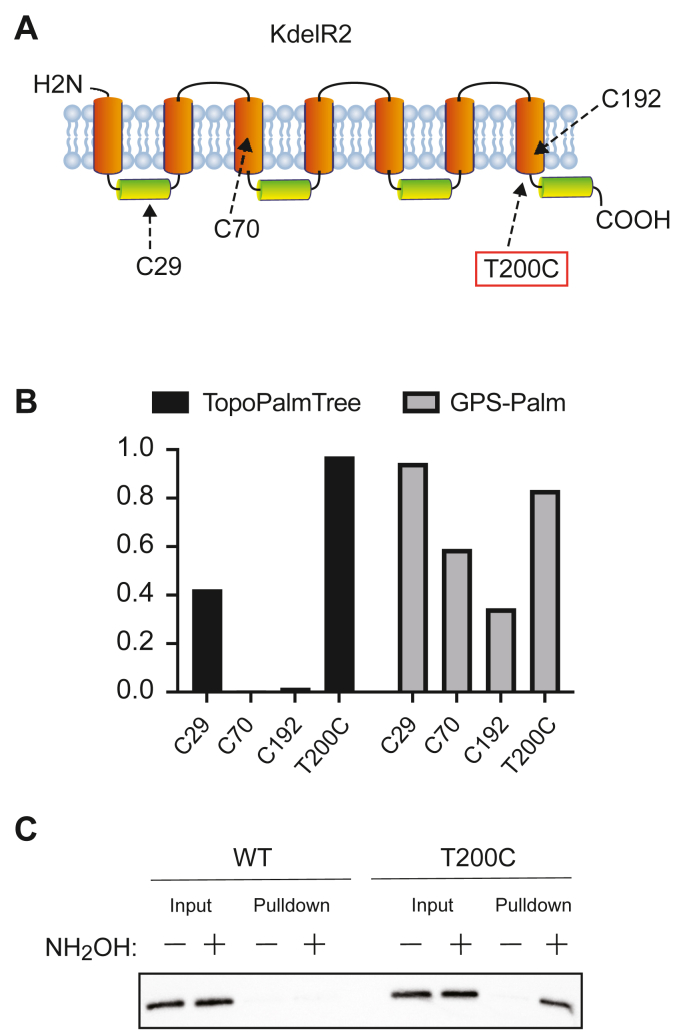
**TopoPalmTree facilitates *in silico* “design” of an *S*-palmitoyl site.***A*, schematic of KdelR2 along with (*B*) probability scores of its three native Cys residues. Shown in red is position 200, which is located in the C-terminal juxtamembrane region where nine residues showed probability scores > 0.90 by *in silico* mutagenesis. *C*, Acyl-RAC of WT *versus* T200 C KdelR2-flag in HEK293 cells (n = 3). Hydroxylamine-dependent pulldown is indicative of protein *S*-acylation status.

Having established the utility of TopoPalmTree to identify sites of *S*-palmitoylation, we next questioned whether TopoPalmTree could be used to design a site of *S*-palmitoylation into a protein that is otherwise not *S*-palmitoylated. To identify a protein with Cys residues that was presumably not *S*-palmitoylated, we first screened for proteins that had at least three Cys residues with probability scores < 0.5 for each. One candidate KDEL Receptor 2 (KdelR2)—a TMP that facilitates retrieval proteins from the Golgi back to the ER—has three Cys residues (Fig. 7*A*) with TopoPalmTree generating very low probabilities for Cys70 and Cys192 (Fig. 7*B*) both of which are located within transmembrane domains. TopoPalmTree provides a low-moderate score for Cys29 (0.42) located in the middle of a cytoplasmic loop. On the contrary, GPS-Palm indicates a high score for Cys29 (0.94) with decreasing scores for Cys70 (0.59) and Cys192 (0.35). When subjected to *in silico* Cys-scanning mutagenesis followed by TopoPalmTree inference, 9 of the 11 locations with probability scores > 0.90 were located on the C-terminal juxtamembrane region and C-terminus (Fig. S6). Noting the rapid increase in TopoPalmTree probability output at the transmembrane—cytoplasmic interface (position 200), a gain of Cys mutation was created in this region and subjected to Acyl-RAC alongside wild-type KdelR2. As shown in Figure 7*C*, the incorporation of a Cys residue at position 200 results in hydroxylamine-dependent pulldown of KdelR2, thus confirming the ability of TopoPalmTree to guide the rational design of an *S*-palmitoyl site.

## Discussion

Protein *S*-palmitoylation is a prevalent hydrophobic modification that imparts hydrophobicity and regulates a diverse array of biological processes. Small-scale studies have consistently observed *S*-palmitoylation at juxtamembrane residues of TMPs, and our findings suggest these sites are incompletely characterized by current experimental techniques due to their inherently hydrophobic locations. To address this methodological gap, a GBT based on simple UniProt-derived features (TopoPalmTree) was created from curated *S*-palmitoyl data, validated on an unrelated dataset of viral *S*-palmitoyl proteins to rigorously assess feature meaningfulness, experimentally applied to identify sites of *S*-palmitoylation and subject a TMP (KdelR2) to *S*-palmitoyl rational design.

Unlike exceedingly complex algorithms, TopoPalmTree uses a relatively straightforward model—gradient-boosted trees—requiring very little computing resources and can be trained in an R environment such as RStudio. Being derived from UniProt, the feature set is readily accessible. Thus, the described topological concepts can be easily implemented and adapted for future *in silico S*-palmitoylation studies and facilitate further expansion of the *S*-palmitoyl toolbox. For example, future directions for TopoPalmTree could include merging with chemoproteomic or alternative PTM datasets to understand the interplay of *S*-palmitoylation with drug binding or other PTMs, respectively. Similar to other machine learning approaches, TopoPalmTree is best considered a hypothesis-generating tool useful for guiding experimental evaluation rather than a confirmatory method.

While the utility of TopoPalmTree has been demonstrated, the approach has several significant limitations. First, TopoPalmTree is restricted to TMPs as the utilized topological feature set is not applicable to soluble (or membrane-associated) proteins. Secondly, TopoPalmTree’s reliance on UniProt-derived topological information means that the model is limited to the number of proteins with available topological data. In the case of the murine proteome, approximately one-quarter of TMPs (1065 total) have incomplete annotation leaving a significant portion of the TMP proteome inaccessible to TopoPalmTree. This limitation can be addressed with continued topological annotation of TMPs. Despite these limitations, TopoPalmTree should provide a valuable resource for future studies on TMP *S*-palmitoylation, particularly those focused on the molecular and functional consequences of this increasingly appreciated post-translational modification.

## Experimental procedures

### Reagents and cell culture

All chemicals and reagents specific to cloning and cell culture are listed in table format in the supporting information. HEK293 cells were cultured in DMEM supplemented with 10% FBS and 100 U/ml penicillin-streptomycin. Cells were grown in a 5% CO2 atmosphere. Transfections were performed for 18 to 24 h using 2.5:1 (μl: μg) ratio polyethyleneimine (PEI) to plasmid in OptiMem (50 μl per 1 μg plasmid). For most experiments, a 6 cm TC-treated plate was transfected with 3 μg of indicated plasmid. SDS-PAGE was performed in 12% acrylamide using the Bio-Rad Mini-Protean System. Transfer to PVDF was performed with the Trans-Blot SD Semi-Dry Transfer Cell (Bio-Rad). Membrane blocking and primary and secondary antibody exposures were performed in TBST containing 5% dried milk (w/v). The primary antibody was incubated overnight. Membranes were visualized with Clarity Western ECL Substrate (Bio-Rad) and a Chemidoc MP imager (Bio-Rad).

### Biophysical characterization of juxtamembrane and swiss palm-derived *S*-palmitoyl sites

The murine proteome from UniProtKB was read into RStudio (version 2023.12.1 + 402). Each cysteine was extracted from the protein sequence into a new row followed by *in silico* trypsinization on the C-terminal side of every Lys or Arg residue except when followed by Pro. All Cys-containing peptides were subjected to molecular weight and mean hydrophobicity measurements based on the Kyte-Doolittle scale (65). Visualizations were performed using ggplot2 and Prism.

### Training dataset and gradient boosted tree

The complete dataset of “Sites” was downloaded from Swisspalm (Version 2022–09–03) and filtered within column “site_techniques” for terms “Point mutation” or “palmitate”. Upon filtering for the seven indicated species and the addition of 22 positive class data corresponding to all sites with palmitoyl fraction > 0.25 from Rodenburg *et al.* (24), stringr package was employed to split topological data into new columns along with character to numeric class conversions. Topological lengths along with relative and absolute distances from the ends of each topology were calculated for every Cys residue. Less common topological locations such as “Lumenal” and “Stromal” were reclassified as “Extracellular” to ensure adequate alignment of categorical variables. The orientation of transmembrane domains (inward *versus* outward) was determined by identifying a row that 1) shared the same accession number and 2) had a stop position that was one integer lower than the transmembrane’s start position. If the preceding domain was identified as “extracellular/lumenal” or “cytoplasmic” the transmembrane domain was considered inward or outward, respectively. Five amino acid windows were extracted on the N- and C-terminal side of each Cys residue along with calculation of Cys scoring, hydrophobicity, charge states, polarity, aliphatic index, and transmembrane tendency were calculated and each was assigned its own column. To ensure complete assignment of the positive class, the SwissPalm-derived *S*-palmitoyl assignments were cross-referenced to UniProt leading to the re-assignment of 17 sites from negative to positive. Upon establishment of the training dataset, the gbm package was utilized for the implementation of a gradient-boosted machine algorithm. The training was configured *via* the caret package. Control parameters included: method = “cv”, number = 10, classProbs = TRUE and summaryFunction = twoClassSummary. After tuning by interaction grid, the final hyperparameters have interacted depth = 18, n.trees = 2000, shrinkage = 0.008 and n.minobsinnode = 5.

### Viral validation dataset

The viral holdout dataset was assembled from three sources with no more than three orthologues per protein type: 27 proteins from UniProtKB, five proteins from the primary literature summarized by Veit (4), and four proteins from SwissPalm verified by radiolabeling or site-directed mutagenesis. Of these 36 proteins, 30 had suitable topographical annotations from UniProt. Following feature extraction, the model was applied to the dataset with probability thresholds of 0.25, 0.5, and 0.75 for binary classification. The same data were exported in FASTA format, submitted to Windows format GPS-Palm in batch format, and read back into Rstudio for analysis.

### Cloning and site-directed mutagenesis

To obtain a cDNA library, RNA was isolated from murine lungs with the RNAeasy kit (Qiagen) followed by reverse transcription with the SuperScript III Reverse Transcription kit (Invitrogen) using oligo-dT primers. DNAs were amplified by PCR and subjected to restriction-ligation into pCMV-EGFP. Site-directed mutagenesis was performed using the NEBasechanger tool and NEB’s Q5 mutagenesis protocol. All relevant oligonucleotide sequences are listed in the supporting information. To generate expression plasmids for Chodl and Havcr2 (including point mutants), genes were synthesized by Twist Biosciences containing C-terminal flag tag and ligated into pCMV-EGFP at the 5′ EcoRI and 3′ NotI sites (replacing the EGFP with the gene of interest). For Cadm4, synthesized genes with C-terminal flag tags were ligated into pCDNA3.1(+) at the 5′ HindIII and 3′ EcoRI sites. All clones were confirmed by Sanger sequencing.

### Annealed oligo ligation

To replace the EGFP with a flag epitope tag, 2 μg of EGFP-containing plasmid was subjected to digestion with AgeI and Not I, and gel extracted using the Qiagen Qiaex II kit. The flag-encoding oligos (100 μM each) were subjected to phosphorylation with T4 PNK according to the NEB protocol at 37 °C for 30 min. After heating to 95 °C for 5 min, annealing was accomplished with a ramp to 25 °C at 5 °C /min. The phosphorylated and annealed oligo was diluted 1:100 into nuclease-free water, then 1 μl was added to a 10 μl T4 ligation reaction including 50 ng of digested vector. After 30 min at room temp, 1 μl of ligation was used to transform chemically competent *E coli*. Colonies were confirmed by Sanger sequencing.

### Assay of *S*-palmitoylation by Acyl-RAC

The Acyl-RAC assay was performed as described (36) with several modifications. For each sample, one 6 cm dish of HEK293 cells or 1 mg of murine lung tissue was subjected to lysis in 100 mM HEPES, 5 mM EDTA, 0.5% Triton X-100, pH 7.2 containing 20 mM N-ethyl maleimide (NEM, made from a fresh 1M stock solution in MeOH). Following probe sonication on ice, lysates were centrifuged at 5000*g* for 10 min and supernatant was transferred into 2 ml blocking reactions containing 100 mM HEPES, 5 mM EDTA, 1% SDS, and 20 mM NEM. Blocking was performed at 50 °C for 1 h with frequent vortexing, then proteins were precipitated with three volumes (6 ml of room temperature MeOH). Samples were mixed, and incubated at −20 °C for 30 min, and pellets recovered with centrifugation at 3000*g* for 5 min. The liquid was aspirated, and the dried pellet was resuspended in 10 ml of MeOH by vortexing (this step ensures complete removal of NEM from the blocking step, which can compete with capture). Protein was again collected with centrifugation at 3000*g* for 5 min, dried, and resuspended in 800 μl of 100 mM HEPES, 5 mM EDTA, 1% SDS pH 7.2. Half of each reaction was transferred onto 25 μl of pyridyl disulfide sepharose (PDS) followed by the addition of either water or 2M neutral NH_2_OH to the final concentration of 500 mM NH_2_OH. Samples were rotated at room temperature for 12 to 18 h, washed three times with 1 ml of wash buffer (50 mM HEPES, 2 mM EDTA, 1% SDS), and eluted with 80 μl of wash buffer containing 20 mM DTT. After 20 min at room temperature, eluant was collected, mixed with 4x Laemlli buffer, heated to 95C for 5 min then analyzed by SDS-PAGE with western blotting. For flag epitope detection, Sigma M2 antibody was used at 1:4000 dilution in TBST containing 5% milk (w/v). A secondary goat anti-mouse HRP antibody (Southern Biotech) was used at 1:2000 dilution in TBST containing 5% milk (w/v). Clarity western ECL substrate (Bio-Rad) was used alongside a ChemiDoc MP Imaging System (Bio-Rad) utilizing standard settings for chemiluminescent and colorimetric (for MW ladder) detections.

## Data availability

All original code has been deposited to Github at https://github.com/mt-forrester/topopalmtree_jbc_2025.

Code is also available at

https://drive.google.com/drive/folders/1FAfElxsRTymUac68VN2UaIT0BWoTy_X1?usp=drive_link.

Please see README.txt for information regarding R markdown and associated csv files.

## Supporting information

This article contains supporting information.

## Conflicts of interest

The authors declare that they have no conflicts of interest with the contents of this article.

## References

[1] Busquets-Hernández C., Triola G. (2021). Palmitoylation as a key regulator of Ras localization and function. Front. Mol. Biosci..

[2] Blaskovic S., Blanc M., van der Goot F.G. (2013). What does S-palmitoylation do to membrane proteins?. FEBS J.

[3] Thorp E.B., Boscarino J.A., Logan H.L., Goletz J.T., Gallagher T.M. (2006). Palmitoylations on murine coronavirus spike proteins are essential for virion assembly and infectivity. J. Virol..

[4] Veit M. (2012). Palmitoylation of virus proteins. Biol. Cell.

[5] Friedman J.H. (2001). Greedy function approximation: a gradient boosting machine. Ann. Stat..

[6] Chen M., Zhang W., Gou Y., Xu D., Wei Y., Liu D. (2023). Gps 6.0: an updated server for prediction of kinase-specific phosphorylation sites in proteins. Nucleic Acids Res..

[7] Poretsky E., Andorf C.M., Sen T.Z. (2023). PhosBoost: improved phosphorylation prediction recall using gradient boosting and protein language models. Plant Direct.

[8] Kroll A., Ranjan S., Lercher M.J. (2024). A multimodal Transformer Network for protein-small molecule interactions enhances predictions of kinase inhibition and enzyme-substrate relationships. PLoS Comput. Biol..

[9] Pradhan U.K., Meher P.K., Naha S., Das R., Gupta A., Parsad R. (2024). ProkDBP: toward more precise identification of prokaryotic DNA binding proteins. Protein Sci..

[10] Dou B., Zhu Z., Merkurjev E., Ke L., Chen L., Jiang J. (2023). Machine learning methods for small data challenges in molecular. Sci. Chem Rev..

[11] Schweizer A., Rohrer J., Kornfeld S. (1995). Determination of the structural requirements for palmitoylation of p63. J. Biol. Chem..

[12] Chamberlain L.H., Shipston M.J. (2015). The physiology of protein S-acylation. Physiol. Rev..

[13] Charollais J., Van Der Goot F.G. (2009). Palmitoylation of membrane proteins (Review). Mol. Membr. Biol..

[14] Oh Y., Jeon Y.J., Hong G.S., Kim I., Woo H.N., Jung Y.K. (2012). Regulation in the targeting of TRAIL receptor 1 to cell surface via GODZ for TRAIL sensitivity in Tumor Cells. Cell Death Differ.

[15] Hayashi T., Rumbaugh G., Huganir R.L. (2005). Differential regulation of AMPA receptor subunit trafficking by palmitoylation of two distinct sites. Neuron.

[16] Lin D.T., Makino Y., Sharma K., Hayashi T., Neve R., Takamiya K. (2009). Regulation of AMPA receptor extrasynaptic insertion by 4.1N, phosphorylation and palmitoylation. Nat. Neurosci..

[17] Kandasamy S.K., Larson R.G. (2006). Molecular dynamics simulations of model trans-membrane peptides in lipid bilayers: a systematic investigation of hydrophobic mismatch. Biophys. J..

[18] Roldan N., Goormaghtigh E., Pérez-Gil J., Garcia-Alvarez B. (2015). Palmitoylation as a key factor to modulate SP-C-lipid interactions in lung surfactant membrane multilayers. Biochim. Biophys. Acta.

[19] Li Y., Pu F., Wang J., Zhou Z., Zhang C., He F. (2021). Machine learning methods in prediction of protein palmitoylation sites: a brief review. Curr. Pharm. Des..

[20] Ning W., Jiang P., Guo Y., Wang C., Tan X., Zhang W. (2021). GPS-Palm: a deep learning-based graphic presentation system for the prediction of S-palmitoylation sites in proteins. Brief Bioinform..

[21] Weng S.L., Kao H.J., Huang C.H., Lee T.Y. (2017). MDD-Palm: identification of protein S-palmitoylation sites with substrate motifs based on maximal dependence decomposition. PLoS One.

[22] Xue Y., Chen H., Jin C., Sun Z., Yao X. (2006). NBA-Palm: prediction of palmitoylation site implemented in Naïve Bayes algorithm. BMC Bioinformatics.

[23] Reddy K.D., Malipeddi J., DeForte S., Pejaver V., Radivojac P., Uversky V.N. (2017). Physicochemical sequence characteristics that influence S-palmitoylation propensity. J. Biomol. Struct. Dyn..

[24] Rodenburg R.N.P., Snijder J., van de Waterbeemd M., Schouten A., Granneman J., Heck A.J.R. (2017). Stochastic palmitoylation of accessible cysteines in membrane proteins revealed by native mass spectrometry. Nat. Commun..

[25] Shipston M.J. (2011). Ion channel regulation by protein palmitoylation. J. Biol. Chem..

[26] Kostiuk M.A., Corvi M.M., Keller B.O., Plummer G., Prescher J.A., Hangauer M.J. (2008). Identification of palmitoylated mitochondrial proteins using a bio-orthogonal azido-palmitate analogue. FASEB J.

[27] Martin B.R., Wang C., Adibekian A., Tully S.E., Cravatt B.F. (2011). Global profiling of dynamic protein palmitoylation. Nat. Methods.

[28] Karthigeyan K.P., Zhang L., Loiselle D.R., Haystead T.A.J., Bhat M., Yount J.S. (2021). A bioorthogonal chemical reporter for fatty acid synthase-dependent protein acylation. J. Biol. Chem..

[29] Martin B.R., Cravatt B.F. (2009). Large-scale profiling of protein palmitoylation in mammalian cells. Nat. Methods.

[30] Yount J.S., Moltedo B., Yang Y.Y., Charron G., Moran T.M., López C.B. (2010). Palmitoylome profiling reveals S-palmitoylation-dependent antiviral activity of IFITM3. Nat. Chem. Biol..

[31] Yount J.S., Zhang M.M., Hang H.C. (2011). Visualization and identification of fatty acylated proteins using chemical reporters. Curr. Protoc. Chem. Biol..

[32] Drisdel R.C., Green W.N. (2004). Labeling and quantifying sites of protein palmitoylation. Biotechniques.

[33] Roth A.F., Wan J., Bailey A.O., Sun B., Kuchar J.A., Green W.N. (2006). Global analysis of protein palmitoylation in yeast. Cell.

[34] Roth A.F., Wan J., Green W.N., Yates J.R., Davis N.G. (2006). Proteomic identification of palmitoylated proteins. Methods.

[35] Forrester M.T., Egol J.R., Tata A., Tata P.R., Foster M.W. (2024). Analysis of protein cysteine acylation using a modified suspension trap (Acyl-Trap). J. Proteome Res..

[36] Forrester M.T., Hess D.T., Thompson J.W., Hultman R., Moseley M.A., Stamler J.S. (2011). Site-specific analysis of protein S-acylation by resin-assisted capture. J. Lipid Res..

[37] Tewari R., West S.J., Shayahati B., Akimzhanov A.M. (2020). Detection of protein S-acylation using acyl-resin assisted capture. J. Vis. Exp..

[38] Giansanti P., Tsiatsiani L., Low T.Y., Heck A.J. (2016). Six alternative proteases for mass spectrometry-based proteomics beyond trypsin. Nat. Protoc..

[39] Jiang Y., Rex D.A.B., Schuster D., Neely B.A., Rosano G.L., Volkmar N. (2024). Comprehensive overview of bottom-up proteomics using mass spectrometry. ACS Meas. Sci. Au..

[40] Tsiatsiani L., Heck A.J. (2015). Proteomics beyond trypsin. FEBS J.

[41] Yates J.R. (2013). The revolution and evolution of shotgun proteomics for large-scale proteome analysis. J. Am. Chem. Soc..

[42] Kar U.K., Simonian M., Whitelegge J.P. (2017). Integral membrane proteins: bottom-up, top-down and structural proteomics. Expert Rev. Proteomics.

[43] Jeffries O., Geiger N., Rowe I.C.M., Tian L., McClafferty H., Chen L. (2010). Palmitoylation of the S0-S1 linker regulates cell surface expression of voltage- and calcium-activated potassium (BK) channels. J. Biol. Chem..

[44] Kuo C.S., Dobi S., Gök C., Da Silva Costa A., Main A., Robertson-Gray O. (2023). Palmitoylation of the pore-forming subunit of Ca(v)1.2 controls channel voltage sensitivity and calcium transients in cardiac myocytes. Proc. Natl. Acad. Sci. U. S. A..

[45] Ulengin-Talkish I., Parson M.A.H., Jenkins M.L., Roy J., Shih A.Z.L., St-Denis N. (2021). Palmitoylation targets the calcineurin phosphatase to the phosphatidylinositol 4-kinase complex at the plasma membrane. Nat. Commun..

[46] Dallavilla T., Abrami L., Sandoz P.A., Savoglidis G., Hatzimanikatis V., van der Goot F.G. (2016). Model-driven understanding of palmitoylation dynamics: regulated acylation of the endoplasmic reticulum chaperone calnexin. PLoS Comput. Biol..

[47] Heo W.D., Inoue T., Park W.S., Kim M.L., Park B.O., Wandless T.J. (2006). PI(3,4,5)P3 and PI(4,5)P2 lipids target proteins with polybasic clusters to the plasma membrane. Science.

[48] Jeffries O., Tian L., McClafferty H., Shipston M.J. (2012). An electrostatic switch controls palmitoylation of the large conductance voltage- and calcium-activated potassium (BK) channel. J. Biol. Chem..

[49] Bélanger C., Ansanay H., Qanbar R., Bouvier M. (2001). Primary sequence requirements for S-acylation of beta(2)-adrenergic receptor peptides. FEBS Lett..

[50] Bizzozero O.A., Bixler H.A., Pastuszyn A. (2001). Structural determinants influencing the reaction of cysteine-containing peptides with palmitoyl-coenzyme A and other thioesters. Biochim. Biophys. Acta.

[51] Grantham R. (1974). Amino acid difference formula to help explain protein evolution. Science.

[52] Zhao G., London E. (2006). An amino acid "transmembrane tendency" scale that approaches the theoretical limit to accuracy for prediction of transmembrane helices: relationship to biological hydrophobicity. Protein Sci..

[53] Blanc M., Blaskovic S., van der Goot F.G. (2013). Palmitoylation, pathogens and their host. Biochem. Soc. Trans..

[54] Abdulrahman D.A., Meng X., Veit M. (2021). S-acylation of proteins of coronavirus and influenza virus: conservation of acylation sites in animal viruses and DHHC acyltransferases in their animal reservoirs. Pathogens.

[55] Blanc M., David F.P.A., van der Goot F.G. (2019). SwissPalm 2: protein S-palmitoylation database methods. Mol. Biol..

[56] Ren J., Wen L., Gao X., Jin C., Xue Y., Yao X. (2008). CSS-Palm 2.0: an updated software for palmitoylation sites prediction. Protein Eng. Des. Sel..

[57] Yadav E., Yadav N., Hus A., Yadav J.S. (2020). Aquaporins in lung health and disease: emerging roles, regulation, and clinical implications. Respir. Med..

[58] King L.S., Agre P. (1996). Pathophysiology of the aquaporin water channels. Annu. Rev. Physiol..

[59] Wang Z., Schey K.L. (2018). Proteomic analysis of S-palmitoylated proteins in ocular lens reveals palmitoylation of AQP5 and MP20. Invest. Ophthalmol. Vis. Sci..

[60] Chang Y., Zhu J., Li X., Deng Y., Lai B., Ma Y. (2024). Palmitoylation regulates myelination by modulating the ZDHHC3-Cadm4 axis in the central nervous system. Signal Transduct. Target Ther..

[61] Sleigh J.N., Barreiro-Iglesias A., Oliver P.L., Biba A., Becker T., Davies K.E. (2014). Chondrolectin affects cell survival and neuronal outgrowth in in vitro and in vivo models of spinal muscular atrophy. Hum. Mol. Genet..

[62] Zhong Z., Ohnmacht J., Reimer M.M., Bach I., Becker T., Becker C.G. (2012). Chondrolectin mediates growth cone interactions of motor axons with an intermediate target. J. Neurosci..

[63] Kuchroo V.K., Meyers J.H., Umetsu D.T., DeKruyff R.H. (2006). TIM family of genes in immunity and tolerance. Adv. Immunol..

[64] Zhang Z., Ren C., Xiao R., Ma S., Liu H., Dou Y. (2024). Palmitoylation of TIM-3 promotes immune exhaustion and restrains antitumor immunity. Sci. Immunol..

[65] Kyte J., Doolittle R.F. (1982). A simple method for displaying the hydropathic character of a protein. J. Mol. Biol..

